# Skin thickness as a potential marker of gestational age at birth despite different fetal growth profiles: A feasibility study

**DOI:** 10.1371/journal.pone.0196542

**Published:** 2018-04-26

**Authors:** Gabriela Luiza Nogueira Vitral, Regina Amélia P. Lopes Aguiar, Ingrid Michelle Fonseca de Souza, Maria Albertina Santiago Rego, Rodney Nascimento Guimarães, Zilma Silveira Nogueira Reis

**Affiliations:** 1 Postgraduation Program of Women’s Health, Faculty of Medicine, Universidade Federal de Minas Gerais, Belo Horizonte, Minas Gerais, Brazil; 2 Department of Gynecology and Obstetrics, Faculty of Medicine, Universidade Federal de Minas Gerais, Belo Horizonte, Minas Gerais, Brazil; 3 Department of Pediatrics, Faculty of Medicine, Universidade Federal de Minas Gerais, Belo Horizonte, Minas Gerais, Brazil; Centre Hospitalier Universitaire Vaudois, FRANCE

## Abstract

**Background:**

New methodologies to estimate gestational age (GA) at birth are demanded to face the limited access to obstetric ultrasonography and imprecision of postnatal scores. The study analyzed the correlation between neonatal skin thickness and pregnancy duration. Secondarily, it investigated the influence of fetal growth profiles on tissue layer dimensions.

**Methods and findings:**

In a feasibility study, 222 infants selected at a term-to-preterm ratio of 1:1 were assessed. Reliable information on GA was based on the early ultrasonography-based reference. The thicknesses of the epidermal and dermal skin layers were examined using high-frequency ultrasonography. We scanned the skin over the forearm and foot plantar surface of the newborns. A multivariate regression model was adjusted to determine the correlation of GA with skin layer dimensions. The best model to correlate skin thickness with GA was fitted using the epidermal layer on the forearm site, adjusted to cofactors, as follows: Gestational age (weeks) = −28.0 + 12.8 *Ln* (Thickness) − 4.4 Incubator staying; *R*^2^ = 0.604 (P<0.001). In this model, the constant value for the standard of fetal growth was statistically null. The dermal layer thickness on the forearm and plantar surfaces had a negative moderate linear correlation with GA (*R* = −0.370, P<0.001 and *R* = −0.421, P<0.001, respectively). The univariate statistical analyses revealed the influence of underweight and overweight profiles on neonatal skin thickness at birth. Of the 222 infants, 53 (23.9%) had inappropriate fetal growths expected for their GA. Epidermal thickness was not fetal growth standard dependent as follows: 172.2 (19.8) μm for adequate for GA, 171.4 (20.6) μm for SGA, and 177.7 (15.2) μm for LGA (P = 0.525, mean [SD] on the forearm).

**Conclusions:**

The analysis highlights a new opportunity to relate GA at birth to neonatal skin layer thickness. As this parameter was not influenced by the standard of fetal growth, skin maturity can contribute to clinical applications.

## Introduction

Noninvasive skin thickness measurement is a medical approach applied in different healthcare areas. Skin image analysis has been supporting innovations in health protection [1, 2] and improvements in cancer research [3]. Diagnostic imaging methods offer a view into skin layers in vivo and in real time. They have advantages over histological methods in that they provide timely results, and morphology maintenance highlights the importance of noninvasive image analyses [2]. High-frequency ultrasonography had a previous validation as a method in determining dermal and subcutaneous thicknesses [4, 5].

Cutaneous aging is a biological phenomenon studied in adults and the elderly. Skin thickness, dermal density, and echogenicity changes are part of the maturity process documented by imaging markers and comparable with histological parameters [6]. During intrauterine life, the skin development process is a continuum that involves embryological steps and maturation of the skin layers, including the stratum corneum [7, 8]. The connection of an age-related morphological postmortem pattern of the fetal skin was previously reported with a high concordance with the chronology of gestation [9]. To date, the relationship between skin layer noninvasive thickening with neonatal age or nutritional status has not been established.

The neonatal age at birth is determined by the chronology of gestation in weeks. The first-trimester obstetric ultrasonography result is the standard that establishes or confirms the gestational age (GA) [10]. However, limited access to obstetric ultrasonography, late prenatal care, and imprecision of postnatal scores of maturity hinders the assessment of the actual GA [11, 12, 13]. Unknown or inaccurate GA results in risks for infants at birth, as caregivers take critical decisions based on the degree of prematurity [14]. Besides, the lack of quality in GA information results in the misclassification of newborn nutrition, resulting in inexact proportions of all the fetal growth profile [15, 16]. New methodologies to estimate GA are claimed by health policies to offer more democratic and easy-to-use solutions, mainly in low- and medium-income countries [11, 12, 17]. This study investigated the correlation between the length of pregnancy and neonatal skin thickness at birth. As secondary objective, it analyzed the influence of underweight and overweight infant profiles on the skin layer dimensions.

## Methods and materials

### Environment and subjects

In this feasibility study, 222 liveborn infants in tertiary referral neonatal care hospitals from January to December 2016 were prospectively selected in accordance with the eligibility criteria. The research protocol was approved by the institutional review boards in Brazil, register No. CAAE 49798915.2.0000.5149. A written informed consent was obtained from each mother on behalf of the newborns. The database is available in S1 Spreadsheet. The inclusion criteria were neonates with GAs of >25 weeks, calculated using an early-ultrasonography-based reference, performed before 14 weeks [10]. Infants with fetal diseases that can affect the skin structure [18], such as fetal hydrops, anhydramnios, or genodermatoses, or clinical evidence of intrauterine infections were excluded. Premature infants were the primary targets during the selection, included at a 1:1 term-to-preterm ratio from an availability sampling. The study size was planned on the basis of a moderate correlation between the GA and the skin thickness (n = 191).

The newborns were classified according to the standard birthweight charts developed by the Intergrowth-21st Study [19, 20, 21]. The 10^th^ and 90^th^ percentiles were considered the lower and upper limits for the definition of small-for-gestational-age (SGA) and Large-for-gestational-age (LGA), the others being classified as Adequate-for-gestational-age (AGA), with birth weights between the 10th and 90th percentiles [22].

Neonatal assessment was performed as soon as possible after birth on the first day of life. The infants were evaluated in the mother-child room or in the neonatal intensive care unit (NICU), inside their incubators or in an open heating crib, wherever they were taken care of to ensure minimum manipulation and stable clinical conditions. A handheld DermaLab USB Series ultrasonography from Cortex Technology scanned the skin over the internal distal forearm and on the plantar posterior surface of the foot once or more until a good-quality image without artifacts was obtained [23]. A mechanical/circular (20 MHz) probe with a resolution of 60 × 200 mm, penetration capacity of 3.4 mm, adjustable gain settling of 10 dB, scan length of 17 mm, and footprint of 11 mm was used. Before data analysis, a final quality review checked the image storage data one by one. Detailed protocol of the skin assessment is available at dx.doi.org/10.17504/protocols.io.nfgdbjw.

### Reliability of measurements

The precision of the thickness measurements was calculated in a sample of 28 volunteer adults aged 18 to 50 years. Abdominal skin image scanning and automated processing using our dedicated software [24] were performed. Precision was defined as the ability to report the same value taken on the same site of the body for several times. The coefficients of variation of 30 assessments per person were calculated, and the precision of the measurements was estimated using twice the standard deviations (SDs) of the mean as the repeatability. The coefficients of variations of epidermal thickness were <1% (mean, 0.34%) for all 28 adults, and the number of their scanned images was 837. The precision of the measurements was 0.114 to 0.187 mm for the images with a 0.150-mm thickness. Besides, the skin was assessed by a second blinded examiner, with a single measurement being acquired by each interrater agreement. The coefficient of variations of epidermal thickness between raters was 1.9% (95% confidence interval [CI] of the mean: 0.7–4.5%).

### Data processing and statistical analyses

**Fig 1:**
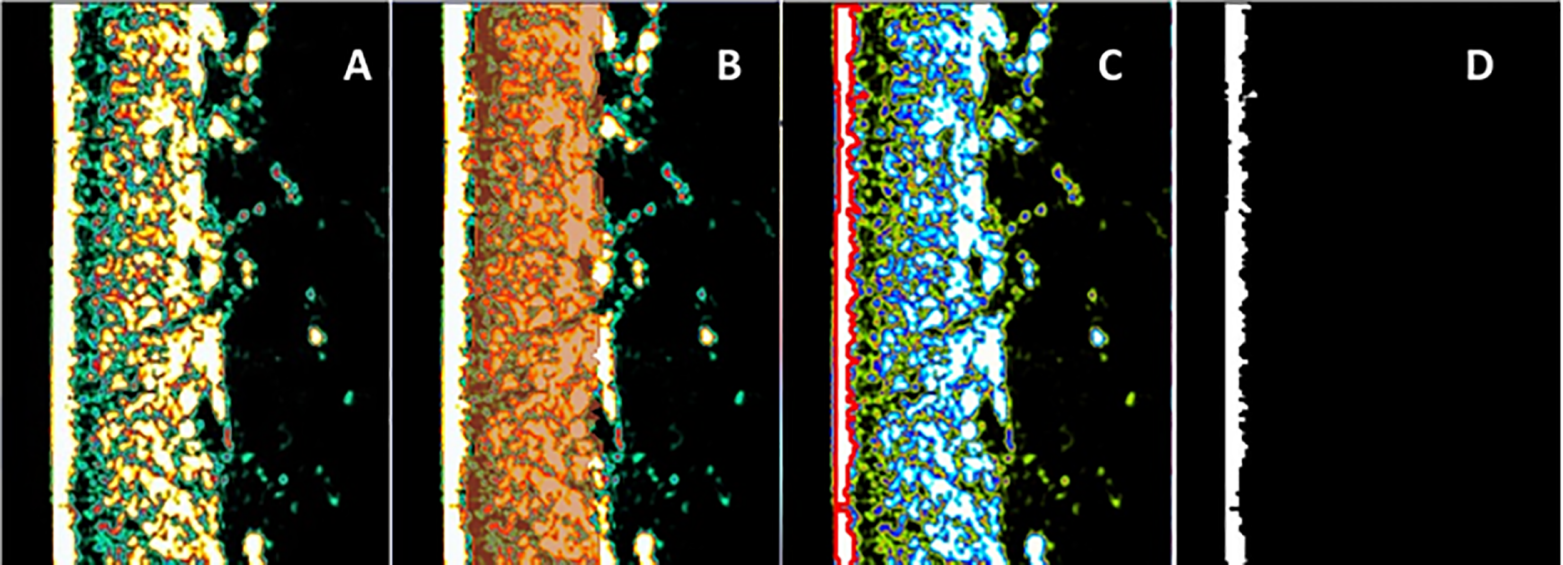
High-frequency ultrasonography image of the skin over the forearm with automated epidermal thickness estimation. (A) Skin ultrasonography image. (B) The red lines and the grid in between indicate the area over which dermal thickness is calculated using the DermaLab^®^ software. (C) The red color corresponds to the automated epidermal boundary detection by our dedicated software. (D) The white and black limits of the epidermis correspond to the automated mean thickness estimation by our dedicated software.

Automated measurements of skin layer thickness involved the use of two different software programs for digital image processing. The DermaLab^®^ embedded software provided dermal measurements (Fig 1A and 1B) but not epidermal thickness. For the epidermal axial dimension estimation, we developed a dedicated software in Python language [24]. The ultrasonography skin image (Fig 1A) with a 356- × 276-pixel resolution automatically identified the epidermis (white layer in Fig 1C, which was separated in a new frame in Fig 1D). The calculation of the epidermal thickness for each line in the image array was based on the size of the pixel in the frame obtaining 356 thickness values. Using a bootstrap technique in the selected sample size with 70 lines, the software resampled the thickness values 2,000 times, obtaining a frequency distribution. From a Gaussian distribution adjustment curve to the epidermis measurement, the peak was the thickness value, and the full width at half-maximum (FWHM) area was the sigma error value. During the image quality analysis, the measurements with non-normal distributions were promptly indicated to promote a visual inspection of the ultrasonography image.

Descriptive statistics was used to assess the clinical, skin measure, and environmental variables during the assessment. Depending on the data distribution, quantitative variables were presented as means (95% CI), standard deviations (SDs), medians (minimum and maximum), or interquartile ranges (IQRs). The magnitude of the skin layer thickness was presented using histograms of frequency. Qualitative variables were presented as absolute values and percentages. The neonatal characteristics and skin layer thickness of the infants were described in accordance with the standard fetal growth (AGA, SGA, and LGA) [21, 22] and compared using Kruskall-Wallis or Chi-square tests.

Growth standard-dependent differences in the skin layer thickness were determined using a one-way analysis of variance or Kruskal-Wallis according to the data distribution and post hoc test. A regression analysis was performed to determine the correlation between GA and skin thickness parameters for each body site. Nonlinear models were adjusted to fit the correlation between predictors and outcomes better. The best body site on the newborn skin to correlate with GA was inferred from the regression coefficients obtained in the scatter plot of skin layer thickness versus GA. Multiple regression analysis included predictor variables from the univariate models, considering the effect modifiers from incubator stay, and the standard fetal growth, using the enter method of model arrangement. Coefficients of determination (adjusted *R*^2^) were determined on the basis of the hypothesis that all coefficients were 0. A normality test for the residual analysis was performed. The statistical program SPSS^®^ 22.0 was used for the analysis. The significance level adjusted for the hypothesis test was set at 5% with 95% CIs.

## Results

### Newborn characteristics

GA ranged from 24 to 41 weeks of gestation. Prematurity occurred in 116 infants (52.3%). Among these infants, 13 (11.2%) were born at GAs of <28 weeks, 23 (19.8%) were born preterm with GAs of 28 to <32 weeks, and 78 (67.2%) were preterm with 32 to <37 weeks. Of the 222 infants, 53 (23.9%) had an inappropriate fetal growth expected for their GA, 36 (16.2%) were underweight, and 17 (7.7%) were overweight. GA at birth and prematurity rate were similar between the groups (Table 1, lines 1 and 4). A maternal nutrition-dependent difference in the neonatal classification of fetal growth, with lower body mass index (BMI) in the SGA group related to AGA (P = 0.039) and LGA infants (P = 0.028; Table 1, line 2). Furthermore, major malformations and longer NICU stays were more frequent in the SGA infants (Table 1, lines 7 and 8).

**Table 1:** Clinical characteristics of the studied gestation and newborns, considering birth weight patterns.

Neonatal and obstetric characteristics	AGA n = 169 (76.1%)	SGA n = 36 (16.2%)	LGA n = 17 (7.7%)	Total n = 222 (100.0%)	P-value
**Gestational age, weeks median (range)**	**36.1 (24.1–41.8)**	**35.2 (26.7–40.5)**	**36.9 (31.6–41.0)**	**37.6 (26.1–41.8)**	**0.275**^a^
**Maternal nutrition (BMI), kg/m2 median (range)**	**29.2 (18.4–42.9)**	**27.2b (18.8–74.2)**	**30.5 (21.8–46.1)**	**28.9 (18.4–74.2)**	**0.014**^a^
**Twinning n (%)**	**22 (13.0)**	**3 (8.3)**	**0**	**25 (11.3)**	**0.224**^d^
**Prematurity n (%)**	**87 (51.5)**	**22 (61.1)**	**7 (42.2)**	**116 (52.3)**	**0.366**^c^
**Birth weight, g median (range)**	**2800.0**^b^ **(525–3990)**	**1752.5**^b^ **(510–2810)**	**3705.0**^b^ **(2025–4340)**	**2680.0 (510–4340)**	**<0.001**^a^
**Male n (%)**	**91 (53.8)**	**19 (52.8)**	**11 (64.7)**	**121 (54.5)**	**0.675**^c^
**Major malformations n (%)**	**8 (4.7)**	**7 (19.4)**	**3 (17.6)**	**18 (8.1)**	**0.004**^d^
**NCIU at the assessment n (%)**	**60 (35.5)**	**21 (58.3)**	**5 (29.4)**	**86 (38.7)**	**0.027**^c^
**Incubator stay n (%)**	**57 (33.7)**	**18 (50.0)**	**5 (29.4)**	**80 (36.0)**	**0.153**^c^

AGA: Appropriate-for-gestational age; LGA: Large-for-gestational age; SGA: Small-for-gestational age; BMI: Body Mass Index; NICU: Neonatal intensive unit care.

^a^Kruskal-Wallis test

^b^Post hoc test significant

^c^Pearson chi-square test

^d^Chi-square test by likelihood ratio

### Skin layer thickness at birth

**Fig 2:**
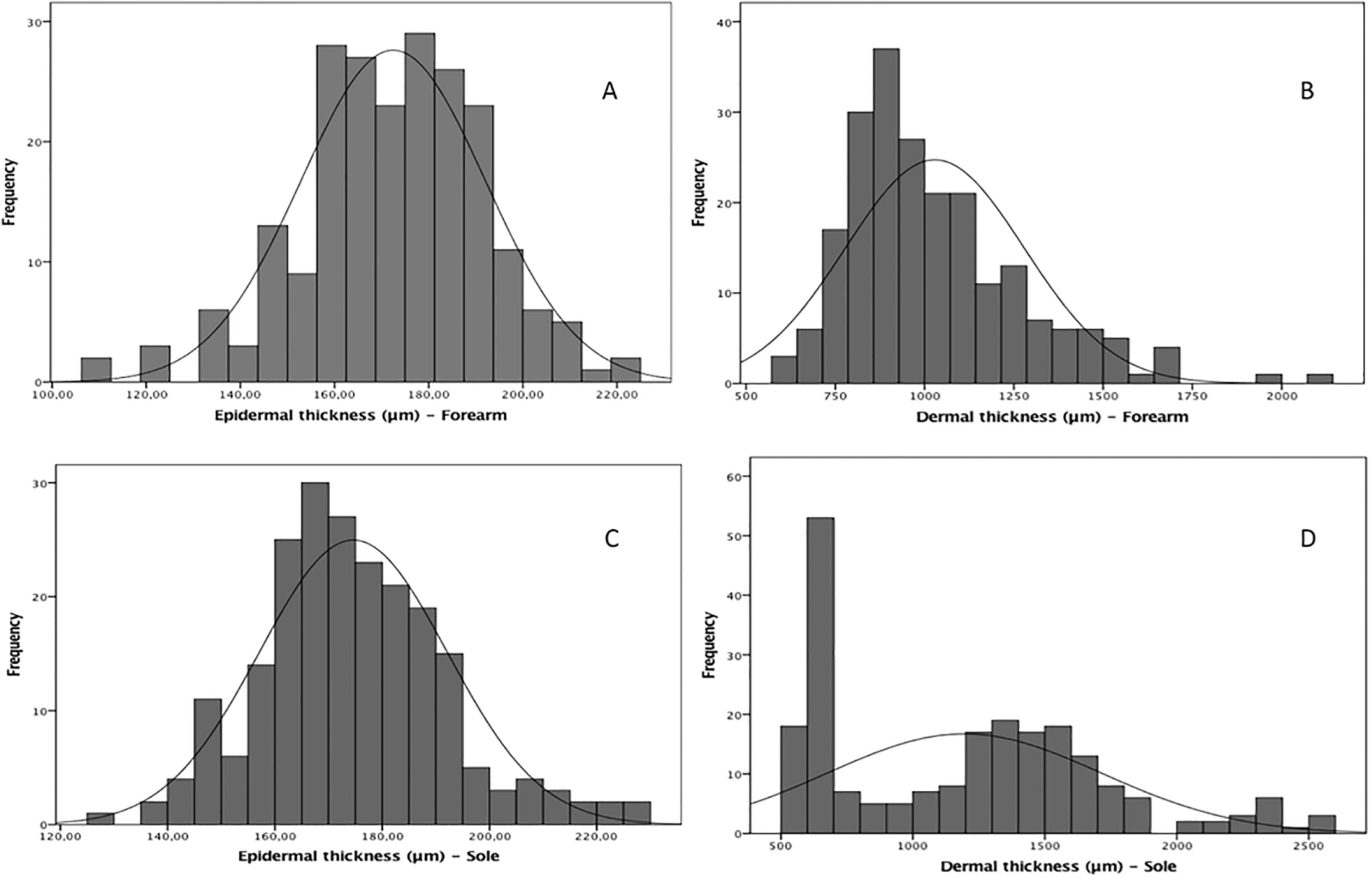
Magnitudes of the skin layer thicknesses of 222 newborns on the first day of life. (A) Distribution corresponds to the epidermal skin over the forearm. Values: n = 217; mean (SD), 172.4 (19.6) μm and 95% CI of the mean, 169.8–175.1. (B) Distribution corresponds to the dermal skin over the forearm. Values: n = 217; mean (SD), 974.0 (290.0) μm; 95% CI of the mean, 994.9–1061.8; and median (IQR), 974.0 (290.0) μm. (C) Distribution corresponds to the epidermal skin over the plantar surface. Values: n = 219; mean (SD), 174.6 (17.5) μm; 95% CI of the mean, 172.2–176.9. (D) Distribution corresponds to the dermal skin over the plantar surface. Values: n = 219; mean (SD), 1244.5 (869.0) μm; 95% CI of the mean, 1125.2–1263.9); and median (IQR), 1244.5 (869.0) μm.

Four hundred thirty-six skin images were selected on the basis of quality after excluding 10 images (2.0%). The magnitudes of the skin thicknesses of the epidermal and dermal layers of the 222 newborns on the first day of life was presented as histograms of frequency (Fig 2). The mean epidermal thickness at the sole had a similar dimension at the forearm as follows: 172.4 (19.6) μm and 174.6 (17.5) μm (P = 0.227). However, the median dermal thickness had a higher dimension at the sole (1244.5 [869.0] μm) than at the forearm (974.0 [290.0] μm; P < 0.001).

### Environmental conditions during the assessment

Some of the newborns (86, 38.7%) were assessed in the NICU under variable conditions of temperature and humidity, depending on the installed interventions such as incubators, equipment, catheters, and medical devices. The other newborns in the maternal-child room received phototherapy inside incubators or in open heating cribs. The environmental conditions during the scanning are presented in Table 2.

**Table 2:** Environmental conditions and neonatal oximetry during the newborn assessment.

Environment parameters	n	Values
Ambient temperature °C, mean (SD)	219	25.7 (2.0)
Ambient humidity %, mean (SD)	221	56.9 (8.7)
Incubator temperature °C, mean (SD)	81	33.7 (1.7)
Incubator humidity %, median (range)	75	53.0 (64)
Newborn temperature* °C, mean (SD)	48	36.7 (1.8)
Oximetry at the beginning of assessment* %, median (IQR)	53	96.0 (1.5)
Oximetry at the ending of assessment* %, median (IQR)	53	96.0 (3.0)

SD: Standard deviation; IQR: Interquartile range

*When under monitoring

### GA prediction based on newborn skin thickness

The epidermal skin thickness on the plantar surface had a weak linear correlation with GA (*R* = 0.150, *R*^2^ = 0.022, P = 0.027). Epidermal skin thickness on the forearm correlated with the gestational length in all the newborns, in the natural logarithm function (*R* = 0.610, *R*^2^ = 0.371, P < 0.001, n = 217). The dermal skin thicknesses on the forearm and plantar surface were found to have a negative linear correlation with GA, with the following values: *R* = −0.370, *R*^2^ = 0.137, P < 0.001 for the forearm site and *R* = −0.421, *R*^2^ = 0.177, P < 0.001 for the plantar surface site.

**Fig 3:**
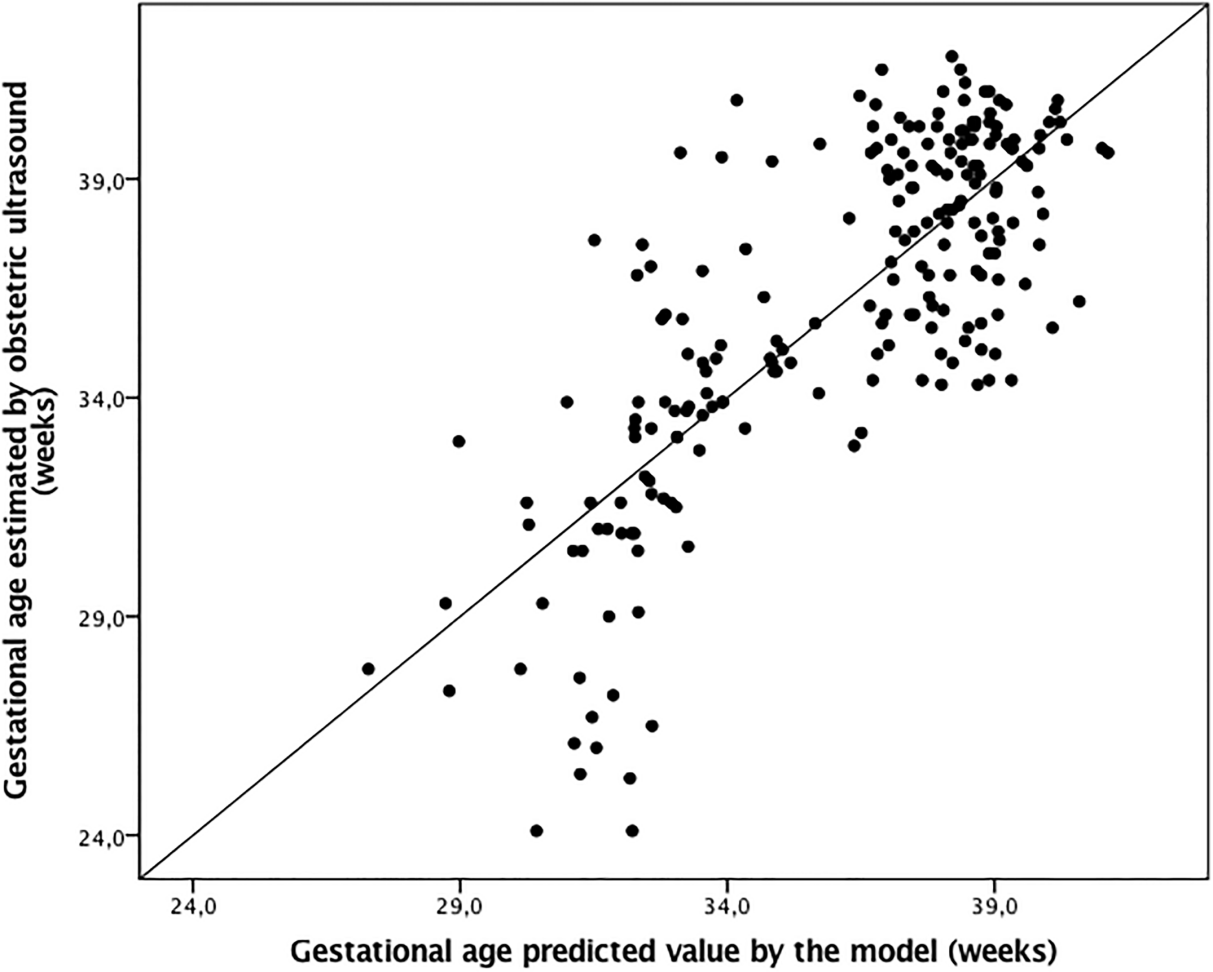
Gestational age estimated using the epidermal skin thickness of the forearm in comparison with GA at birth by early obstetric ultrasonography. Equation: Gestational age (weeks) = −28.0 + 12.8 *Ln* (Thickness) − 4.4 Incubator stay + 0.015 standard fetal growth; *R* = 0.777, *R*^2^ = 0.604, P < 0.001.

The best adjusted multivariate model to explain the correlation between skin epidermal thickness and GA was that which included the epidermal layer on the forearm, adjusted by the incubator staying cofactor as follows: Gestational age (weeks) = −28.0 + 12.8 *Ln* (Epidermal thickness) − 4.4 Incubator staying; *R*^2^ = 0.604, P < 0.001. In this multivariate model, the constant value for the standard fetal growth was 0.015 (P = 0.993). Another constant had a P value of <0.001 for individual nullity test. Predicted values versus GA estimated using obstetric ultrasonography had a correlation of *R* = 0.78 (P < 0.001; Fig 3).

**Fig 4:**
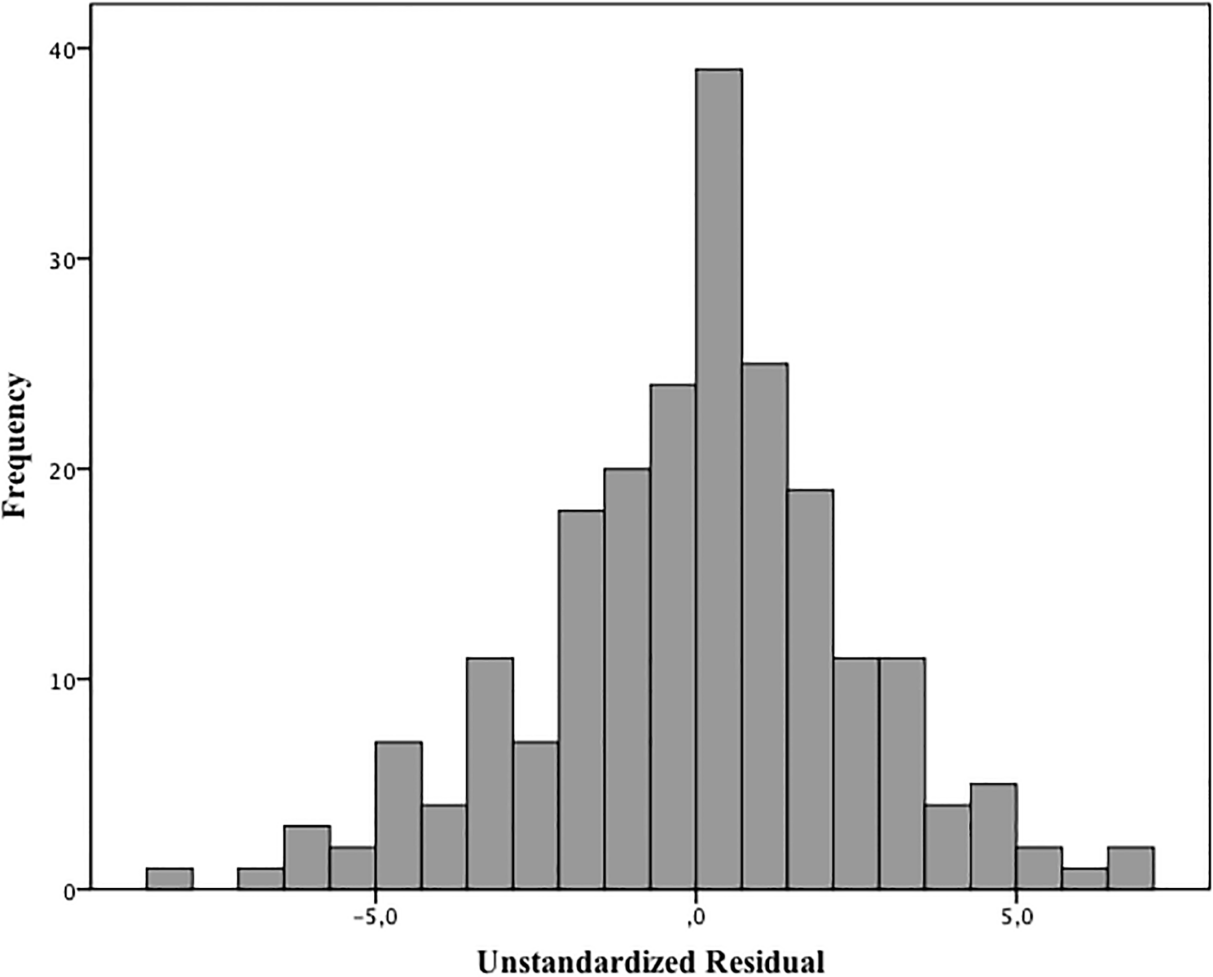
Histogram of residual value for the skin thickness vs. gestational age.

The residual GA values that were not explained by the model had a normal distribution and estimated error of 17 days for the GA (Kolmogorov-Smirnov test, P = 0.092 Fig 4). It means that 95% of occasions, GA calculated by the model did not differ more than 34 days from GA estimated by obstetric ultrasonography (Fig 4).

### The influence of standards of fetal growth on the neonatal skin layers dimensions

Epidermal thickness had a normal distribution, and the mean (SD) dimensions were similar among the three groups, considering the skin on the forearm and plantar surface (Table 3, lines 2 and 7). Dermis, dermis-to-epidermis ratio, and total skin had asymmetrical distributions and were presented as medians and IQRs. No birthweight pattern-dependent differences in the skin layer thickness of the dermis on the forearm (P = 0.301) and plantar surface (P = 0.692), despite the asymmetrical trimodal distribution of the latter data (Fig 2D). No median (IQR) differences in the dermis-to-epidermis ratio or total skin thickness on the forearm and plantar surface (Table 3).

**Table 3:** Variation of skin thickness at birth according to birthweight patterns and examination site in newborns.

	AGA	SGA	LGA	Total	P-value
	Mean (SD) Median (IQR)	Mean (SD) Median (IQR)	Mean (SD) Median (IQR)	Mean (SD) Median (IQR)	
**Skin over forearm**	**n = 166**	**n = 35**	**n = 16**	**n = 217**	
**Epidermis (μm)**	**172.2 (19.8)**	**171.4 (20.6)**	**177.7 (15.2)**	**172.4 (19.6)**	**0.525**^a^
**Dermis (μm)**	**948.0 (293.0)**	**1053.0 (316.0)**	**948.0 (277.0)**	**974.0 (290.0)**	**0.301**^b^
**Dermis-to-epidermis ratio**	**5.5 (2.2)**	**6.0 (2.2)**	**5.6 (1.7)**	**5.6 (2.2)**	**0.362**^b^
**Total skin (μm)**	**1123.2 (256.6)**	**1248.3 (321.3)**	**1098.6 (292.6)**	**1153.1 (260.4)**	**0.252**^b^
**Skin over plantar face**	**n = 166**	**n = 36**	**n = 17**	**n = 219**	
**Epidermis (μm)**	**173.9 (17.5)**	**176.5 (18.1)**	**177.1 (17.0)**	**174.6 (17.5)**	**0.631**^a^
**Dermis (μm)**	**1264.0 (882.0)**	**1198.0 (809.0)**	**1099.0 (836.0)**	**1244.5 (869.0)**	**0.692**^b^
**Dermis-to-epidermis ratio**	**7.4(5.3)**	**6.4 (5.2)**	**4.5 (5.5)**	**6.8 (5.2)**	**0.619**^b^
**Total skin (μm)**	**1430.1 (894.9)**	**1369.1 (858.9)**	**1037.7 (833.7)**	**1390.8 (873.4)**	**0.695**^b^

AGA: Appropriate for gestational age; LGA: Large-for-gestational age; SGA: Small-for-gestational age; SD: Standard deviation; IQR: Interquartile range.

^a^ANOVA

^b^Kruskal-Wallis test

## Discussion

### Main findings of the study

In this paper, we first present the potential of a new approach to estimate indirectly the GA at birth based on ultrasonography biometric measurements of the skin layers. The noninvasive epidermal skin measurement on the forearm correlated with the gestation length, mirroring the intrauterine stratification and keratinization of the surface responsible for tissue thickening, related to skin maturation. A new opportunity to infer the GA at birth based on the epidermal thickness emerged. As a feasibility study, the results highlighted whether the intervention is appropriate for further well-controlled testing.

The primary motivation of this study is the importance of accurate GA determination in reducing prematurity-related adverse outcomes in a birth setting without this critical information. Despite the established gold standard for GA [10], not all pregnant women needing opportune ultrasonography could access it [12]. Neonatal assessment of GA based on maturity scores has presented wide margins of error [13]. In low- and medium-income countries, lack of obstetric ultrasonography access is a concern [12] and accurate gestation chronology data are frequently unavailable [25]. Unplanned pregnancy and late prenatal care are situations not exclusive to resource-constrained countries [26], which challenge advances and new GA markers at birth.

On the basis of the gold standard assessment tool for determining GA using a noninvasive postnatal method, such outcomes can influence the search for postnatal gestational length markers. However, accurate estimation of GA at birth greatly varies because of the fetal intrinsic growth potentials and maternal influences on the intrauterine nutrition, resulting in inexact due dates. For instance, maternal diseases complicating pregnancy [27], socioeconomic status [28], and fetal malformations impact fetal nutrition and functional maturation of systems [29, 30].

Hidden prematurity is the clinical target that needs to be achieved, considering that high-risk newborns are often affected by intrauterine growth disturbances. Regarding this aspect, our results can detect the lack or small effect of fetal growth input on skin thickening. The birth-weight patterns did not influence the epidermal and dermal thicknesses. This might be because the dimensions did not include the subcutaneous fat layer. Skin development has a layered organization, including the avascular epidermis and vascularized dermis [18, 31]. The epidermal layer is essentially formed by keratinocytes from 20 weeks of gestation and becomes functional late in fetal life [32]. Neonatal skin contains less total lipids than adult skin [33].

**Fig 5:**
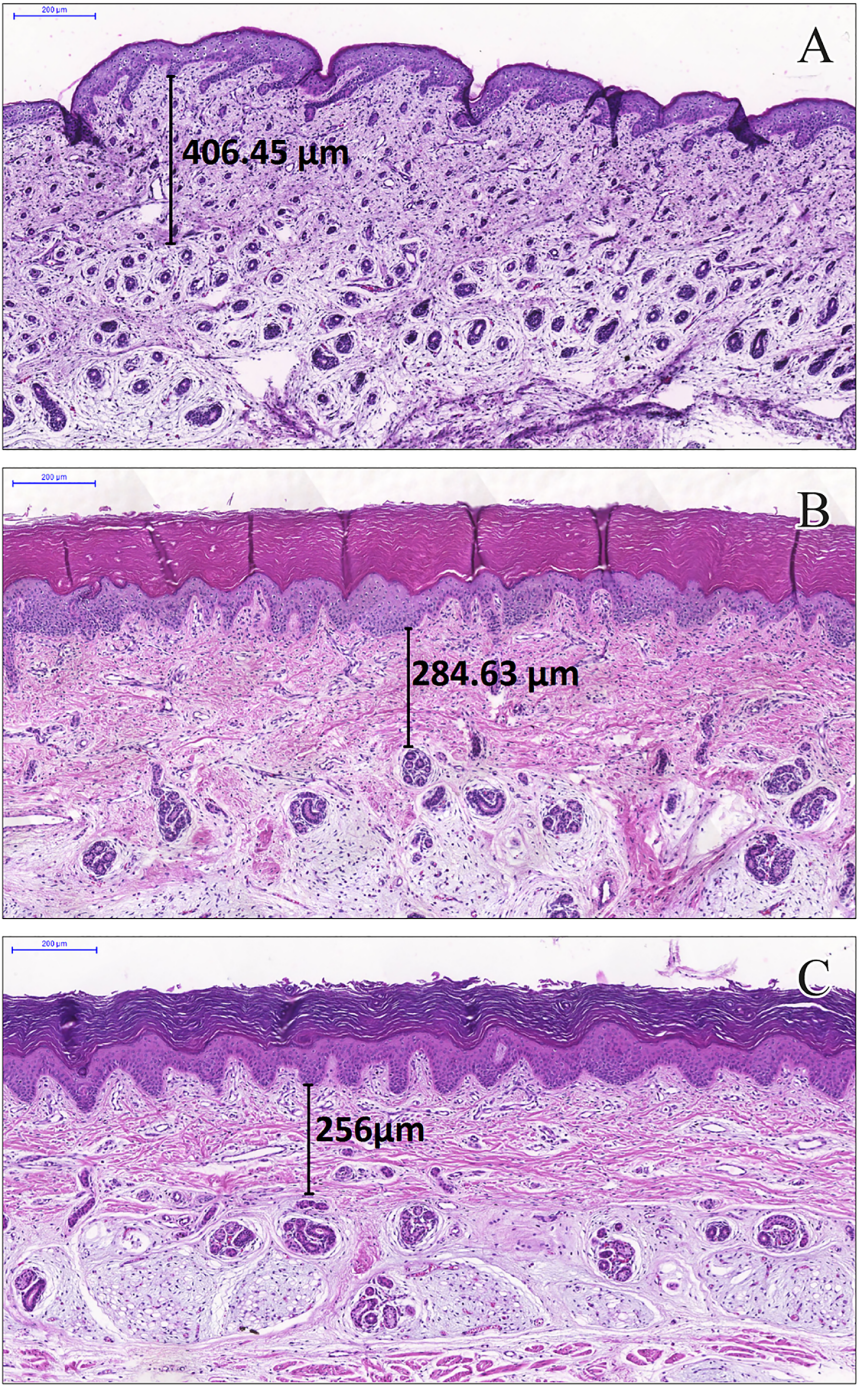
Neonatal skin with dermal layer measurements in stillbirths from biopsies over the plantar surface of the foot. Scale: 200 μm. Skin at (A) 21 weeks of gestation, 406.5 μm; (B) 34 weeks of gestation, 284.6 μm; and (C) 40 weeks of gestation.

The intriguing result could not be explained with our proposal and data. The negative correlation between the dermal layer thickness and GA, though weak, deserves more attention and further investigation. Skin anchoring evolution in the dermis and lipid skin barrier over the epidermis are related to expressive structural modifications [34] that can be associated with progressive dermal changes. To support this reasoning, we used the skin images obtained at 21, 34, and 40 weeks of gestation, when the dermal layer was more defined and less thick than at term (Fig 5).

### Comparison of the skin measurements with those in previous studies

Skin thickness analysis based on noninvasive assessments to verify the intrauterine fetal growth effects is a novel approach. Few reports have been conducted on noninvasive neonatal skin thickness measurement, mainly including premature infants. A pioneer study on neonatal skin that used a high-frequency ultrasonography evaluated the relationship of the dermal and subcutaneous fat thickness to the skinfold measure to support the nutritional evaluation in the neonatal period [35]. In this study, we found that the skin thickness magnitude on the plantar surface was comparable with that in the study by Petersen et al., in which they used an equivalent image acquisition but only in seven newborns [35]. Other studies reported the anatomical variability of skin thickness to support vaccine delivery in 384 children after 4 months of age [36] and 10 children in another study to verify the effects of fitness on the skin [4]. Physiological skin structure modifications throughout the lifespan included skin thickness in 42 infant and adult subjects [37]. Despite GA influence on the epidermal thickness in our sample, the skin layer dimensions were comparable with the previous findings.

The comparability and credibility of our results can be attributed to the learning curve of our team (GLNV and IMFS) before the newborn assessments were conducted to determine the repeatability control and automated measurements of epidermal and dermal thicknesses via digital image processing software. High-risk newborn evaluations demand previous trainings and rigid procedure standardizations to avoid contamination and manipulation risks during the ultrasonographic examination to obtain reliable data. Environmental control (particularly temperature) during newborn assessment was a concern, as internal body heat can affect skin thickness during physical activity [37]. These conditions were stable during the assessments. Skin thickness measurement performed using high-frequency ultrasonography has previously shown precision, reproducibility, and validation as a method in determining only dermal and subcutaneous thicknesses in various anatomical locations [4, 5, 37], combined with gold standard methods [3, 38, 39]. We tested our own measurement repeatability for epidermal thickness, which corroborated previous reliabilities for the dermal and subcutaneous layers.

### Strengths and limitations

The asymmetrical dermal dimension distributions taken on the plantar surface suggests a possible bias. This finding is expected, as the examiners noticed plantar reflex stimulation when the probe with a jelly touched the skin over the foot during the assessment, as expected in newborns [18]. We attribute the absence of linear and nonlinear correlations with GA in this site to unpredictable plantar contraction variations, which affect the skin elasticity during in vivo analyses. Furthermore, dermal detection limits via high-frequency ultrasonography in extremely premature infants weighing 510 g, as in our sample, were not reported. The dermal layer did not appear to be as well defined as the epidermal layer on the premature infant images and may not have been adequately measured using the equipment-embedded software.

The outcomes in this study should be interpreted with caution. The high-risk pregnancy rate represents referrals to neonatal services, not the general newborn population. Furthermore, low birth weight, intrauterine growth restriction, and prematurity are complex and heterogeneous antenatal health problems with multifactorial etiologies [28, 29] that may not be entirely represented in our sample, which limits the generalizability of our results. The expected fetal growth is a marker of health of pregnant women and long-term adverse consequences reflected in the compositions of neonatal tissues, organs, and functions [29, 40, 41]. Conclusions from this analysis were derived from overall findings from the whole study sample. Further analyses using other study designs that focus on each fetal growth abnormalities are still necessary to confirm our initial interpretation. Refinements of the multivariate model based on a larger sample size and various GAs are still needed before clinical proposals can be made.

Moreover, our research group reported that neonatal skin reflectance, adjusted by clinical variables, was related to GA, with higher correlation than skin thickness (*R*^2^ = 0.828, P < 0.001) [42]. However, both analyses revealed bio-optical properties of the skin at birth as a target to improve maturity scores of GA prediction.

### Conclusions

We emphasize skin thickness as a newborn maturity marker at birth, without the influence of the fetal growth standard. Newborn weight (underweight or overweight) has a potential to be added to the neonatal maturity assessment tool and GA prediction. Nevertheless, we still believe that skin maturity, reflected in the skin thickness dimension, can contribute to clinical applications that aid in improving the classification of neonates into groups with low birth weight, prematurity, and intrauterine growth restriction, which are directly linked to accurate prediction of GA.

## Supporting information

S1 Spreadsheet Excel spreadsheet presenting unidentified clinical data and skin thickness measurement from the acquisitions. (PDF)

## Abbreviations

AGA: adequate for gestational age
BMI: body mass index
CI: confidence interval
FWHM: full with a half-maximum
GA: gestational age
IQR: interquartile range
LGA: large for gestational age
NICU: neonatal intensive care unit
SD: standard deviation
SGA: small for gestational age

## References

[1] Lambert PH, Laurent PE. Intradermal vaccine delivery: will new delivery systems transform vaccine administration? Vaccine 2008;26: 3197–3208. doi: 10.1016/j.vaccine.2008.03.095 18486285 10.1016/j.vaccine.2008.03.095

[2] Sattler E, Kästle R, Welzel J. Optical coherence tomography in dermatology. J Biomed Opt 2013;18: 061224–061224. doi: 10.1117/1.JBO.18.6.061224 23314617 10.1117/1.JBO.18.6.061224

[3] Meyer N, Lauwers-Cances V, Lourari S, Laurent J, Konstantinou MP, Lagarde JM, et al High-frequency ultrasonography but not 930-nm optical coherence tomography reliably evaluates melanoma thickness in vivo: a prospective validation study. Br J Dermatol 2014;171: 799–805. doi: 10.1111/bjd.13129 24863700 10.1111/bjd.13129

[4] Schou A, Thomsen K, Plomgaard A, Wolthers O. Methodological aspects of high-frequency ultrasound of skin in children. Skin Res Technol 2004;10: 200–206. doi: 10.1111/j.1600-0846.2004.00070.x 15225271 10.1111/j.1600-0846.2004.00070.x

[5] Tan C, Statham B, Marks R, Payne P. Skin thickness measurement by pulsed ultrasound; its reproducibility, validation and variability. Br J Dermatol 1982;106: 657–667. 7082570 10.1111/j.1365-2133.1982.tb14702.x

[6] Crisan D, Lupsor M, Boca A, Crisan M, Badea R. Ultrasonographic assessment of skin structure according to age. Indian J DermatolVenereol Leprolol 2012;78: 519. 10.4103/0378-6323.98096 22772636

[7] Moll I, Moll R, Franke WW. Formation of epidermal and dermal merkel cells during human fetal skin development. J Investig Dermatol 1986;87: 779–787. 3782861 10.1111/1523-1747.ep12458993

[8] Hardman MJ, Moore L, Ferguson MW, Byrne C. Barrier formation in the human fetus is patterned. J Investig Dermatolo 1999;113: 1106–1113. 10.1046/j.1523-1747.1999.00800.x 10594759

[9] Ersch J, Stallmach T. Assessing gestational age from histology of fetal skin: an autopsy study of 379 fetuses. Obstet Gynecol 1999;94: 753–757. 10546723 10.1016/s0029-7844(99)00379-8

[10] Committee opinion No. 611: method for estimating due date. Obstet Gynecol 2014;124: 863–866. doi: 10.1097/01.AOG.0000454932.15177.be 25244460 10.1097/01.AOG.0000454932.15177.be

[11] Lee AC, Mullany LC, Ladhani K, Uddin J, Mitra D, Ahmed P, et al Validity of newborn clinical assessment to determine gestational age in Bangladesh. Pediatrics 2016;138: e20153303 doi: 10.1542/peds.2015-3303 27313070 10.1542/peds.2015-3303 PMC4925072

[12] Karl S, Suen CSLW, Unger HW, Ome-Kaius M, Mola G, White L, et al Preterm or not–an evaluation of estimates of gestational age in a cohort of women from rural Papua New Guinea. PloS One 2015;10: e0124286 https://doi.org/10.1371/journal.pone.0124286. 25945927 10.1371/journal.pone.0124286 PMC4422681

[13] Lee AC, Panchal P, Folger L, Whelan H, Whelan R, Rosner B, et al Diagnostic accuracy of neonatal assessment for gestational age determination: a systematic review. Pediatrics 2017;140(6): e20171423 doi: 10.1542/peds.2017-1423 29150458 10.1542/peds.2017-1423

[14] Mason E, McDougall L, Lawn JE, Gupta A, Claeson M, Pillay Y, et al From evidence to action to deliver a healthy start for the next generation. Lancet 2014;384: 455–467. doi: 10.1016/S0140-6736(14)60750-9 24853599 10.1016/S0140-6736(14)60750-9

[15] Wingate MS, Alexander GR, Buekens P, Vahratian A. Comparison of gestational age classifications: date of last menstrual period vs. clinical estimate. Ann Epidemiol 2007;17: 425–430. doi: 10.1016/j.annepidem.2007.01.035 17395481 10.1016/j.annepidem.2007.01.035

[16] Pereira APE, Dias MAB, Bastos MH, do Carmo Leal M. Determining gestational age for public health care users in Brazil: comparison of methods and algorithm creation. BMC Res Notes 2013;6:60 doi: 10.1186/1756-0500-6-60 23402277 10.1186/1756-0500-6-60 PMC3585703

[17] Adeyekun A, Orji M. Predictive accuracy of transcerebellar diameter in comparison with other foetal biometric parameters for gestational age estimation among pregnant Nigerian women. East Afr Med J 2014;91: 138–144. 26859033

[18] Oranges T, Dini V, Romanelli M. Skin physiology of the neonate and infant: clinical implications. Adv Wound Care 2015;4: 587–595. doi: 10.1089/wound.2015.0642 26487977 10.1089/wound.2015.0642 PMC4593874

[19] Villar J, Ismail LC, Victora CG, Ohuma EO, Bertino E, Altman DG, et al International standards for newborn weight, length, and head circumference by gestational age and sex: the Newborn Cross-Sectional Study of the INTERGROWTH-21st Project. Lancet 2014;384: 857–868. doi: 10.1016/S0140-6736(14)60932-6 25209487 10.1016/S0140-6736(14)60932-6

[20] Villar J, Papageorghiou AT, Pang R, Ohuma EO, Ismail LC, Barros FC, et al The likeness of fetal growth and newborn size across non-isolated populations in the INTERGROWTH-21st Project: the Fetal Growth Longitudinal Study and Newborn Cross-Sectional Study. Lancet Diabetes Endocrinol 2014;2: 781–792. doi: 10.1016/S2213-8587(14)70121-4 25009082 10.1016/S2213-8587(14)70121-4

[21] Villar J, Giuliani F, Fenton TR, Ohuma EO, Ismail LC, Kennedy SH et al INTERGROWTH-21^st^ very preterm size at birth reference charts. Lancet 2016;387(10021): 844–845. doi: 10.1016/S0140-6736(16)00384-6 26898853 10.1016/S0140-6736(16)00384-6

[22] INTERGROWTH-21st calculator. [cited 2016 Aug 12 [Internet] http://intergrowth21.ndog.ox.ac.uk/en/ManualEntry/Compute.

[23] Cortex Technology. DermaLab^®^ Series SkinLab USB Instruction Manual. In: [Cortex Technology] Book DermaLab^®^ Series SkinLab USB Instruction Manual. Hadsund: Cortex Technology; 2015. 41 pages.

[24] Vitral G, Fonseca M, Magalhães W, Guimarães R, Reis Z. Determinação automática da espessura da epiderme em imagens ultrassonográficas In: Sociedade Brasileira de Informática em Saúde editors. Book Determinação automática da espessura da epiderme em imagens ultrassonográficas. Sociedade Brasileira de Informática em Saúde: Goiânia, 2016.

[25] Graafmans WC, Richardus JH, Macfarlane A, Rebagliato M, Blondel B, Verloove-Vanhorick SP, et al Comparability of published perinatal mortality rates in Western Europe: the quantitative impact of differences in gestational age and birthweight criteria. BJOG 2001;108: 1237–1245. 11843385 10.1111/j.1471-0528.2001.00291.x

[26] Wellings K, Jones KG, Mercer CH, Tanton C, Clifton S, Datta J, et al The prevalence of unplanned pregnancy and associated factors in Britain: findings from the third National Survey of Sexual Attitudes and Lifestyles (Natsal-3). Lancet 2013;382: 1807–1816. doi: 10.1016/S0140-6736(13)62071-1 24286786 10.1016/S0140-6736(13)62071-1 PMC3898922

[27] Catov J, Abatemarco D, Althouse A, Davis EM, Hubel C. Patterns of gestational weight gain among overweight and obese women related to small-and large-for-gestational-age births. Obesity (Silver Spring, Md) 2015;23: 1071. 10.1002/oby.21006 PMC4414675 25865858

[28] Currie J, Moretti E. Biology as destiny? Short-and long-run determinants of intergenerational transmission of birth weight. J Labor Econ 2007;25: 231–264.

[29] Pollack RN, Divon MY. Intrauterine growth retardation: definition, classification, and etiology. Clin Obstet Gynecol 1992;35: 99–107. 1544253 10.1097/00003081-199203000-00015

[30] Robert MF, Neff RK, Hubbell JP, Taeusch HW, Avery ME. Association between maternal diabetes and the respiratory-distress syndrome in the newborn. N Engl J Med 1976;294: 357–360. doi: 10.1056/NEJM197602122940702 1246288 10.1056/NEJM197602122940702

[31] Fluhr J, Darlenski R, Lachmann N, Baudouin C, Msika P, De Belilovsky C, et al Infant epidermal skin physiology: adaptation after birth. Br J Dermatol 2012;166: 483–490. doi: 10.1111/j.1365-2133.2011.10659.x 21967466 10.1111/j.1365-2133.2011.10659.x

[32] Telofski LS, Morello AP, Mack Correa MC, Stamatas GN. The infant skin barrier: can we preserve, protect, and enhance the barrier? Dermatol Res Prac 2012;2012: 198789. 10.1155/2012/198789 PMC3439947 22988452

[33] Agache P, Blanc C, Barrand C, Laurent R. Sebum levels during the first year of life. Br J Dermatol 1980;103: 643–650. 7459260 10.1111/j.1365-2133.1980.tb01686.x

[34] Smith LT, Holbrook KA, Byers PH. Structure of the dermal matrix during development and in the adult. J Investig Dermatol 1982;79: 93–104. 10.1111/1523-1747.ep12545877 7086196

[35] Petersen JR, Petersen S, Serup J. High-frequency ultrasound measurement of dermis and subcutaneous fat in the newborn infant. Skin Res Technol 1995;1: 86–89. doi: 10.1111/j.1600-0846.1995.tb00023.x 27328388 10.1111/j.1600-0846.1995.tb00023.x

[36] Ploin D, Schwarzenbach F, Dubray C, Nicolas JF, Goujon C, Trong MD, et al Echographic measurement of skin thickness in sites suitable for intradermal vaccine injection in infants and children. Vaccine 2011;29: 8438–8442. doi: 10.1016/j.vaccine.2011.07.111 21821081 10.1016/j.vaccine.2011.07.111

[37] Seidenari S, Giusti G, Bertoni L, Magnoni C, Pellacani G. Thickness and echogenicity of the skin in children as assessed by 20-MHz ultrasound. Dermatology 2000;201: 218–222. doi: 10.1159/000018491 11096192 10.1159/000018491

[38] Vyas S, Meyerle J, Burlina P. Non-invasive estimation of skin thickness from hyperspectral imaging and validation using echography. Comput Biol Med 2015;57: 173–181. doi: 10.1016/j.compbiomed.2014.12.010 25561244 10.1016/j.compbiomed.2014.12.010

[39] Rippon M, Springett K, Walmsley R, Patrick K, Millson S. Ultrasound assessment of skin and wound tissue: comparison with histology. Skin Res Technol 1998;4: 147–154. doi: 10.1111/j.1600-0846.1998.tb00101.x 27328910 10.1111/j.1600-0846.1998.tb00101.x

[40] Young MF, Nguyen PH, Addo OY, Pham H, Nguyen S, Martorell R, et al Timing of gestational weight gain on fetal growth and infant size at birth in Vietnam. PloS One 2017;12: e0170192 doi: 10.1371/journal.pone.0170192 28114316 10.1371/journal.pone.0170192 PMC5256875

[41] Veena SR, Krishnaveni GV, Karat SC, Osmond C, Fall CH. Testing the fetal overnutrition hypothesis; the relationship of maternal and paternal adiposity to adiposity, insulin resistance and cardiovascular risk factors in Indian children. Public Health Nutr 2013;16: 1656–1666. doi: 10.1017/S1368980012003795 22895107 10.1017/S1368980012003795 PMC3622715

[42] Reis ZSN, Vitral GLN, de Souza IMF, Rego MAS, Guimaraes RN. Newborn skin reflection: Proof of concept for a new approach for predicting gestational age at birth. A cross-sectional study. PLOS ONE. 2017;12(9):e0184734 https://doi.org/10.1371/journal.pone.0184734. 28931040 10.1371/journal.pone.0184734 PMC5607181

