# Supplementary material for: Skin thickness as a potential marker of gestational age at birth despite different fetal growth profiles: A feasibility study
Source: PLoS One. 2018 Apr 26;13(4):e0196542. doi: 10.1371/journal.pone.0196542 (PMC5919437; doi:10.1371/journal.pone.0196542)
Supplement: S1 Spreadsheet — (PDF) [file pone.0196542.s001.pdf]

IG_GOLD	BIRTHWEIGTH	EPIDERMIS_THICKNESS_FOREARM(	DERMIS_THICKNESS_FOREARM(	EPIDERMIS_THICKNESS_SOLE(	DERMIS_THICKNESS_SOLE(	TWINNING
24,1	625	136,5	1514	146,4	1053	0
24,1	525	157,1	1172	164,8	964	0
25,3	705	156,6	1317	157,6	1040	1
25,4	845	145,6	2133	136,8	1277	1
26,0	935	149,0	1290	162,4	1396	1
26,1	840	144,3	1211	148,4	1317	0
26,5	920	161,7	1119	188,1	2344	0
26,7	715	148,3	1053	139,1	790	0
27,2	910	152,8	1475	166,5	1725	0
27,3	510	120,3	1132	172,9	742	0
27,6	885	145,4	1146	176,6	1527	0
27,8	1170	133,4	1290	166,7	1527	0
27,8	1250	106,7	1093	175,8	961	0
29,0	1209	151,8	1132	160,4	1435	1
29,0	1136			173,5	1304	1
29,1	825	158,7	922	181,5	2225	1
29,2	835			166,7	777	0
29,3	1165	137,7	1527	167,4	1606	1
29,3	1240	119,5	1409	171,4	1159	1
30,5	925	144,3	1514	168,9	1448	0
30,5	1235	158,4	1554	209,6	2146	1
30,5	1130	146,0	908	189,0	1698	1
30,6	1115	170,5	1343	169,2	1106	0
30,9	1535	154,7	1172	183,5	1488	0
30,9	1215	156,9	1001	186,9	1106	0
30,9	1195	112,0	1369	157,1	1422	0
31,0	1560	151,5	1698	166,8	1224	1
31,0	1310	149,5	1945	163,4	1435	1
31,1	1505	135,0	1475	166,1	1277	0
31,5	1085	167,7	961	163,4	1224	0
31,6	2025	166,1	1475	173,5	2449	0
31,6	1535	134,6	1698	169,1	2502	0

31,6	2850	154,3	882	193,8	1053	0
31,6	890	147,9	948	176,2	895	0
31,7	1115	164,7	1620	174,0	816	0
31,8	1750	161,6	1396	209,4	1435	0
32,1	1750	161,1	658	166,1	1698	0
32,2	1855	160,0	1132	180,8	1448	0
32,8	2815	173,1	1080	175,0	1593	0
32,9	1205	154,7	1277	176,6	1540	0
33,0	1575	121,8	619	158,7	1119	0
33,1	1290	168,0	1277	167,5	1185	0
33,1	925	168,0	948	149,6	1488	1
33,1	1930	157,7	843	155,0	1606	1
33,2	2470	156,2	1488	169,8	632	0
33,3	2090	157,6	856	178,1	1791	0
33,3	2445	161,3	750	146,6	1304	0
33,3	2020	185,3	1264	195,7	1883	0
33,5	2405	157,8	1462	199,2	658	0
33,6	2020	174,1	895	183,5	1580	0
33,7	1900	170,1	1422	185,0	816	0
33,7	1085	167,4	672	172,0	1040	0
33,8	2195	170,6	764	165,2	1843	1
33,8	1890	176,7	1659	181,2	1870	1
33,9	2020	142,7	1435	172,2	1856	0
33,9	2335	179,3	764	193,5	1330	1
33,9	1680	158,5	1422	183,9	1001	1
33,9	2055	179,4	1066	181,7	1343	0
33,9	1755	164,8	1106	189,1	1159	1
34,1	1780	175,2	1066	175,4	1027	0
34,1	2170	146,7	777	169,5	1264	0
34,3	2285	185,2	908	159,5	1501	0
34,3	2305	175,6	1251	175,0		0
34,4	2505	188,3	803	162,0	1396	0
34,4	2590	170,7	882	173,0	1501	0

34,4	1850	158,8	816	180,1	1488	0
34,4	2300	194,6	698	161,5	1343	0
34,6	1535	194,3	961	210,6	1606	0
34,6	2515	175,0	922	156,9	2080	0
34,6	2670	137,4	882	145,7	1304	0
34,8	1105	198,4	1304	205,7	830	0
34,8	1980	174,2	1396	158,2	1527	0
34,8	3015	178,3	1238	156,8	1224	0
34,8	1545	137,2	1685	158,6	2080	0
34,9	3300	192,0	961	173,2	1791	0
34,9	1905	177,6	724	178,1	1527	0
35,0	1680	170,6	750	157,1	988	0
35,0	2190	159,9	830	146,7	2317	0
35,0	2670	175,5	882	185,6	1435	0
35,0	2635	190,1	843	186,7	645	0
35,1	2690	195,8	895	165,3	1554	0
35,1	2600	186,2	764	190,5	1580	0
35,2	1415	162,7	750	163,4	1396	0
35,2	2300	178,9	1014	196,0	2330	0
35,3	1895	138,1	1238	165,2	1830	0
35,3	2575	181,8	974	193,8	592	0
35,6	2300	173,1	816	162,6	1778	0
35,6	2885	182,7	737	162,6	1264	0
35,6	2415	206,6	685	169,1	1053	0
35,7	2650	186,1	1080	174,2	1580	0
35,7	2625	145,9	830	161,8	1488	0
35,7	2643	161,0	908	173,5	1488	0
35,8	2740	164,1	859	160,2	1290	0
35,8	2095	169,3	1093	187,3	2528	0
35,9	2520	164,9	1159	178,8	2120	1
35,9	1830	168,9	619	149,7	922	0
35,9	2765	161,9	1066	163,1	2357	1
35,9	2730	167,7	988	178,3	1409	1

35,9	2670	190,7	830	173,0	2554	0
36,0	2165	176,4	1080	222,3	1290	0
36,1	2300	173,3	1001	181,0	2212	0
36,1	3080	158,1	895			0
36,2	2330	214,9	895	175,0	1606	0
36,3	2950	190,5	935	206,5	619	0
36,3	2745	172,5	869	166,9	1672	0
36,6	2460	198,5	1080	157,2	1514	0
36,7	2800	190,8	816	176,8	1633	0
36,7	2560	163,7	895	213,3	579	0
36,8	2630	186,1	658	146,6	1211	0
36,8	3410	172,1	935	163,5	1843	0
36,8	2780	177,8	908	185,5	1672	0
36,8	2460	158,2	922	161,0	1264	0
36,9	2680	184,9	988	172,8	1685	0
36,9	3185	174,1	922	183,3	1659	0
37,0	1655	161,6	1514	178,8	1211	0
37,0	3265	170,6	1053	189,1	592	0
37,1	2605	163,1	856	164,9	1778	0
37,3	2145	190,1	1238	191,5	2330	0
37,3	3090	188,3	724	169,5	1382	0
37,4	1825	185,7	869	184,9	1448	0
37,5	3090	159,4	816	149,5	658	0
37,5	2820	202,7	830	163,6	1462	0
37,5	3450	176,1	606	194,7	843	0
37,6	2885			180,0	619	1
37,6	2825	166,4	988	220,0	672	1
37,6	2975	148,6	1093	156,0	1527	0
37,6	3215	191,2	895	178,9	1343	0
37,6	4005			173,7	1382	0
37,7	4040	186,0	777	169,3	658	0
37,8	2830	168,7	1132	148,2	672	0
37,8	3110	190,8	1172	194,8	764	0

37,8	3195	164,2	948	163,0	1330	0
38,0	2470	184,5	1106	202,5	2225	0
38,0	2465	195,3	1053	195,3	1277	0
38,0	2465	177,4	988	191,6	658	0
38,0	2680	172,0	869	169,6	1317	0
38,1	3000	189,3	843	172,9	606	0
38,1	3705	153,2	908	189,9	619	0
38,2	3280	203,8	908	202,7	645	0
38,2	3110	175,1	908	171,1	1606	0
38,3	3135	178,5	790	178,6	579	0
38,3	3500	177,2	750	194,9	592	0
38,4	3465	180,2	935	189,3	579	0
38,5	2960	164,9	1053	181,3	592	0
38,5	3610	180,7	843	168,3	1238	0
38,7	4295	190,0	1106	213,7	685	0
38,7	3570	202,3	988	184,5	1580	0
38,8	3990	168,3	1027	178,1	632	0
38,8	2815	168,1	1172	191,9	606	0
38,8	3285	190,3	869	160,7	619	0
38,9	2830	184,5	750	187,8	1764	0
39,0	2890	162,7	1146	142,9	1330	0
39,1	3005	185,8	935	175,6	632	0
39,1	2835	177,0	1119	169,5	632	0
39,1	3050	182,4	1001	217,3	1501	0
39,1	2985	164,7	1027			0
39,2	2945	162,2	882	148,1	658	0
39,2	2505	174,6	869	180,5	606	1
39,3	3300	199,1	764	170,5	672	0
39,3	2640	173,4	1093	161,6	606	0
39,3	2700	184,2	935	181,0	592	0
39,3	3315	185,3	843	140,6	645	0
39,3	2800	168,1	988	127,7	1251	0
39,4	2686	193,0	658	188,3	645	0

39,4	3190	197,7	922	191,0	1198	0
39,4	3685	180,9	988	181,5	658	0
39,5	3685	179,1	1238	183,3	579	0
39,6	3530	168,6	790	171,5	606	0
39,6	3125	178,0	988			0
39,6	3465	166,1	1185	169,6	672	0
39,6	3465	158,4	895	160,1	592	0
39,6	2670	224,4	974	171,4	1725	0
39,7	3505	194,9	1066	150,0	632	0
39,7	3045	194,7	988	183,2	685	0
39,7	3190	159,7	1198	189,9	592	0
39,7	3085	202,7	895	175,6	592	0
39,7	3155	222,2	830	156,5	632	0
39,8	2215	193,6	1159	158,8	606	0
39,8	3390	172,1	764	226,5	619	0
39,8	3403	188,5	856	176,6	1751	0
39,8	2645	181,2	1080	196,8	2370	0
39,8	3115	147,0	882	185,5	619	0
39,9	3400	163,2	1027	150,6	1672	0
39,9	3380	177,6	974	192,4	579	0
39,9	2725	183,4	1224	161,7	606	0
39,9	3995	195,1	803	183,9	645	0
39,9	3460	183,7	830	174,7	658	0
39,9	2970	211,1	843	166,4	592	0
40,0	3300	190,2	1224	168,4	724	0
40,0	3860	203,0	974	164,6	645	0
40,1	3465	181,6	856	171,0	606	0
40,1	3225	180,7	1001	171,1	645	0
40,2	3365	174,5	1027	192,7	724	0
40,2	3055	184,3	882	154,9	1396	0
40,2	3385	167,5	843	173,2	619	0
40,2	3780	158,8	1238	172,4	1475	0
40,2	3180	190,4	1356	222,1	579	0

40,2	3375	170,0	1027	229,5	1330	0
40,3	3490	188,5	843	153,4	645	0
40,3	3005	183,9	1343	159,5	606	0
40,3	3300	184,6	948	180,9	1554	0
40,3	3655	209,0	816	168,3	619	0
40,3	3270	205,7	948	186,6	1264	0
40,4	2810	165,5	764	182,9	961	0
40,5	3180	174,8	856	165,7	685	0
40,5	2200	188,8	1014	179,2	672	0
40,6	2975	207,5	803	175,2	1475	0
40,7	3350			144,4	619	0
40,7	3990	193,1	922	162,5	1290	0
40,7	3485	159,5	803	185,2	658	0
40,8	3070	183,1	1080	166,0	777	0
40,8	4290	208,0	882	152,1	685	0
40,8	4115	181,3	1106	178,9	619	0
40,8	3160	191,2	1080	203,8	1119	0
40,9	3595	155,8	895	164,6	619	0
41,0	3005	176,1	1238	170,6	1343	0
41,0	4340	188,2	1001	192,7	672	0
41,0	3420	187,1	777	141,9	1567	0
41,2	3280	181,8	1264	194,2	592	0
41,5	2985	180,6	961	165,8	606	0
41,5	3470	160,9	908	187,8	579	0
41,8	3095	178,3	1001	175,2	592	0

MAJOR_MALFORMATION	NEONATAL_SEX	INTERGROWTH_CLASSIFICATION	NICU_STAY_AT_ASSESSMENT	AMBIENT_TEMPERATURE_C	AMBIENT_HUMIDITY%
0	1	2	1	24,2	51
0	1	2	1	24,6	66
0	1	2	1	24,1	40
0	2	2	1	25,2	44
0	2	2	1	22,7	59
0	2	2	1	24,1	62
0	2	2	1	24,1	56
0	2	1	1	27,0	59
0	1	2	1	24,8	67
0	1	1	1	24,0	50
0	1	2	1		
0	2	2	1	23,2	53
0	1	2	1	26,5	56
0	1	2	1	24,1	51
0	1	2	1	24,1	51
0	2	1	1	24,7	57
0	2	1	1	24,1	56
0	2	2	1	26,7	50
0	2	2	1	26,9	54
0	2	1	1	23,3	52
0	1	2	1	24,2	64
0	1	2	1	24,2	64
0	1	2	1	24,5	55
0	1	2	1	22,8	63
0	1	2	1	25,4	57
0	1	2	1	27,1	54
0	2	2	1	25,1	53
0	2	2	1	25,2	58
0	1	2	1	25,1	50
0	1	1	1	24,3	58
1	1	3	1	23,4	60
0	2	2	1	24,4	52

1	2	3	1	24,7	46
0	1	1	1	27,2	54
0	1	1	1	24,1	53
0	2	2	1	22,8	53
0	1	2	1	25,2	54
0	2	2	1	25,1	52
0	2	3	1	23,6	60
1	2	1	1	23,3	65
1	1	2	1	22,0	61
0	2	1	1	23,9	49
0	2	1	1	24,5	68
0	2	2	1	24,5	68
0	2	2	0	25,2	65
0	2	2	1	23,7	50
0	1	3	1	24,4	58
0	2	2	1	25,4	37
1	2	2	1	21,8	60
0	2	2	1	24,6	57
0	2	2	1	21,9	73
1	2	1	1	23,8	54
0	2	2	1	23,6	55
0	1	2	1	23,8	63
0	1	2	1	23,0	64
0	2	2	1	23,7	72
0	2	2	1	23,8	67
0	2	2	1	23,9	67
0	1	2	1	24,3	64
0	2	2	1	22,8	37
0	1	2	1	24,8	52
0	2	2	0	25,0	63
0	2	2	0	26,3	59
0	2	2	1	22,7	47
0	1	2	0	25,5	61

0	1	2	0	25,7	61
0	1	2	0	27,1	60
0	2	1	1	23,2	49
0	2	2	1	23,8	56
0	2	2	0	29,0	42
0	1	1	1	24,5	57
0	2	2	1	24,8	60
0	2	3	0	25,6	60
0	2	1	0	26,6	52
0	2	3	1	23,9	67
0	1	2	1	24,5	66
1	2	1	1		
0	1	2	0	24,8	58
0	2	2	0	25,5	61
0	2	2	0	26,7	58
0	2	2	1	23,8	66
0	2	2	0	26,3	59
0	1	1	1	26,2	51
0	2	2	1	27,0	46
0	1	1	1	26,7	51
0	1	2	0	27,0	53
0	2	2	0	23,4	52
1	1	2	0	23,7	66
0	2	2	0	25,9	58
0	1	2	0	24,4	56
0	2	2	0	25,0	63
0	2	2	0	26,2	60
0	2	2	1	23,7	73
1	2	1	1	23,9	60
0	2	2	1	23,9	67
0	1	1	0	24,2	39
0	1	2	0	26,0	58
0	1	2	0	26,0	58

0	1	2	0	29,5	54
0	2	1	0	27,4	55
0	1	2	0	23,7	48
0	2	2	0	27,3	57
0	2	2	0	26,2	53
1	2	2	1	23,5	58
0	2	2	0	27,0	58
0	2	2	0	21,9	58
0	1	2	0	25,4	54
0	2	2	0	27,6	53
0	1	2	0		
0	1	3	0	22,0	50
0	2	2	1	24,4	65
1	1	2	1	26,8	47
0	2	2	0	24,1	56
0	2	2	1	26,7	60
0	1	1	1	23,7	72
0	1	2	0	25,3	62
0	1	2	0	24,1	46
0	2	1	0	22,8	49
0	1	2	0	25,0	65
0	1	1	1	23,8	74
0	2	2	0	26,8	58
0	2	2	0	27,6	60
1	1	3	0	27,7	59
0	1	2	0	23,0	60
0	1	2	0	23,0	60
0	2	2	1	23,7	56
0	1	2	0	26,1	58
0	2	3	0	29,0	56
0	1	3	0	27,0	57
0	1	2	0	25,3	59
0	2	2	0	27,3	56

0	1	2	0	30,0	47
1	1	1	0	26,5	59
0	2	1	0	26,8	59
0	1	1	0	26,9	57
1	2	2	0	29,0	56
0	2	2	0	27,3	57
0	2	3	0	31,0	42
0	2	2	0	26,8	56
0	2	2	0	31,0	42
0	2	2	0	25,3	62
0	2	2	0	29,0	56
0	1	2	0	26,8	65
0	2	2	0	26,0	58
0	2	2	0	26,2	53
0	2	3	0	26,3	54
0	2	2	0	26,8	56
0	2	3	0	26,0	58
0	2	2	0	27,0	70
0	2	2	0	27,6	54
0	2	2	0	26,5	58
0	2	2	0	26,5	65
0	2	2	0	25,0	71
0	1	2	0	25,4	62
0	2	2	0	28,0	58
0	1	2	0	28,0	56
0	2	2	0	25,7	59
1	1	1	0	28,0	58
0	2	2	0	26,5	58
0	1	1	0	26,7	60
0	1	2	0	26,8	59
0	2	2	0	27,0	57
0	1	2	0	28,0	58
1	1	1	1	23,9	68

0	2	2	0	26,3	59
0	1	2	0	27,5	60
1	2	2	1	21,8	63
0	2	2	1	22,0	67
0	2	2	0	25,6	60
0	1	2	0	28,0	56
0	1	2	0	28,0	56
0	2	1	0	29,0	42
0	2	2	0	26,0	56
0	1	2	0	26,5	58
0	1	2	0	26,6	56
0	1	2	0	27,2	57
0	2	2	0	29,0	56
0	2	1	0	24,2	60
0	1	2	0	26,5	65
0	2	2	0	26,8	59
0	2	1	0	30,0	50
0	1	2	0	30,0	43
0	1	2	0	26,0	58
0	2	2	0	26,5	65
0	2	1	0	27,0	60
0	2	3	0	27,7	60
0	1	2	0	29,0	51
0	1	2	0	30,0	50
0	2	2	0	24,3	61
0	2	2	0	27,4	61
0	1	2	0	26,7	60
0	2	2	0	28,0	62
0	1	2	0	23,0	60
0	1	2	0	25,5	57
0	1	2	0	26,7	61
0	1	2	0	27,0	70
0	2	2	0	29,0	56

0	1	2	0	29,0	67
0	1	2	0	26,8	59
0	1	2	0	26,8	59
0	1	2	0	27,5	60
0	2	2	0	28,0	58
0	2	2	0	31,0	42
0	1	1	0	29,0	42
0	1	2	0	27,2	57
0	1	1	0	27,4	53
0	2	2	0	27,0	53
0	1	2	0	25,6	60
0	2	2	0	26,0	55
0	1	2	0	27,0	61
1	1	2	1	22,8	53
0	2	3	0	26,0	53
0	2	3	0	26,5	65
0	1	2	0	26,5	59
0	2	2	0	25,0	71
0	1	2	0	26,8	70
0	1	3	0	30,0	50
0	2	2	0	30,0	50
0	1	2	0	26,7	61
0	1	2	0	25,0	62
0	1	2	0	25,4	62
0	2	2	0	24,5	54

INCUBATOR_TEMPERATURE	HUMIDITY_INCUBATOR%	TEMPERATURE_NEWBORN_C	FOTOTERAPY	HEAT_CRIB
35,6	86,0	37,0	0	0
34,6	85,0	36,0	0	0
35,9	70,0	37,2	0	0
35,4	88,0	36,8	1	0
33,9	94,0	36,7	0	0
35,0	82,0	37,1	1	0
33,7	85,0		0	0
34,9	75,0		0	0
34,7	70,0		1	0
36,7	79,0	36,8	0	0
33,7	67,0		1	0
34,9	82,0		0	0
34,1	62,0	36,6	0	0
32,9	76,0	37,1	1	0
31,0	89,0	37,2	1	0
34,0	67,0		0	0
34,0	80,0		1	0
34,0	44,0	37,1	0	0
34,5	70,0		0	0
34,5	73,0	37,0	0	0
34,5	81,0		0	0
34,6	71,0		0	0
34,0	65,0		0	0
33,8	61,0	35,7	0	0
34,8	43,0	36,7	1	0
33,4	76,0	36,5	0	0
32,6	52,0	37,0	0	0
34,4		37,7	0	0
33,5	66,0		1	0
35,5	77,0		0	0
32,9	40,0		0	0
31,8	71,0		0	0

29,9	51,0	37,5	0	0
38,5	39,0	37,7	0	0
33,9	77,0	37,6	0	0
32,0	69,0	37,2	0	0
			1	0
37,0	30,0	36,9	0	0
		38,3	0	0
34,7	35,0		0	0
33,0		37,4	0	0
35,2	48,0	36,4	0	0
33,5	67,0		0	0
31,5	53,0		0	0
			0	0
			0	0
32,2	42,0	37,3	0	0
34,8	39,0	36,5	0	0
33,9	40,0	36,8	0	0
32,0			0	0
34,4	37,0	36,6	0	0
35,5	54,0	36,8	0	0
33,5	42,0	36,4	0	0
34,0	51,0	36,4	0	0
37,6	65,0	36,6	0	0
			0	0
34,0	63,0	37,0	0	0
32,9	41,0	37,1	0	0
33,2	44,0	36,7	0	0
31,6	37,0		0	0
30,0		36,5	0	1
			0	0
			0	0
32,5	44,0		0	0
			0	0

			0	0
			0	0
33,9	40,0		0	0
31,0	39,0		0	0
			0	0
35,9	55,0	37,0	0	0
33,0		36,4	0	0
			0	0
			0	0
32,9	42,0		0	0
32,0	46,0		0	0
33,5	46,0	36,6	0	0
			0	0
			0	0
			0	0
33,8	40,0	37,3	1	0
			0	0
36,5			0	0
35,4	39,0		0	0
30,0	49,0		0	1
			0	0
			0	0
			0	0
			0	0
			0	0
			0	0
			0	0
34,9	42,0	36,2	0	0
32,5	38,0	36,6	0	0
32,5	43,0		0	0
			0	0
			0	0
			0	0

			0	0
			0	0
			0	0
			0	0
			0	0
34,4	37,0	36,7	0	0
			0	0
			0	0
			0	0
			0	0
			0	0
			0	0
36,5	40,0	24,4	1	1
31,9	62,0		0	0
			0	0
32,1	57,0		0	0
33,8	41,0	37,8	0	0
			0	0
			0	0
			0	0
			0	0
34,0	37,0	37,2	0	0
30,6	51,0		1	0
			0	0
			0	0
			0	0
			0	0
31,4	53,0		0	0
			0	0
			0	0
			0	0
			0	0
			0	0

			0	0
			0	0
			0	0
			0	0
			0	0
			0	0
			0	0
			0	0
			0	0
			0	0
			0	0
			0	0
			0	0
			0	0
			0	0
			0	0
			0	0
			0	0
			0	0
			0	0
			0	0
			0	0
			0	0
			0	0
			0	0
			0	0
			0	0
			0	0
			0	0
			0	0
			0	0
			0	0
		36,5	0	0

			0	0
			0	0
33,3	40,0		0	0
			0	0
			0	0
			0	0
			0	0
			0	0
			0	0
			0	0
			0	0
			0	0
			0	0
			0	0
			0	0
			0	0
			0	0
			0	0
			0	0
			0	0
			0	0
			0	0
			0	0
			0	0
			0	0
			0	0
			0	0
			0	0
			0	0
			0	0
			0	0
			0	0
			0	0

			0	0
			0	0
			0	0
			0	0
			0	0
			0	0
			0	0
			0	0
			0	0
			0	0
			0	0
			0	0
			0	0
33,7	37,0		0	0
			0	0
			0	0
			0	0
			0	0
			0	0
			0	0
			0	0
			0	0
			0	0
			0	0
			0	0
